# Hyponatremia and Its Correlation With Hepatic Encephalopathy and Severity of Liver Disease

**DOI:** 10.7759/cureus.13175

**Published:** 2021-02-06

**Authors:** Amna Younas, Junaid Riaz, Tamoor Chughtai, Hamza Maqsood, Muhammad Saim, Shaheryar Qazi, Shifa Younus, Umar Ghaffar, Muhammad Khaliq

**Affiliations:** 1 Medicine, Russells Hall Hospital, Dudley, GBR; 2 Medicine, Nishtar Medical University, Multan, PAK; 3 Cardiology, Nishtar Medical University, Multan, PAK; 4 Internal Medicine, Nishtar Medical University, Multan, PAK

**Keywords:** hyponatremia, liver cirrhosis, hepatic encephalopathy, liver, hepatitis b, hepatitis c

## Abstract

Background and objective

Hepatic cirrhosis is one of the leading causes of morbidity and mortality worldwide. Patients with cirrhosis frequently develop complications such as ascites, variceal bleeding, and hepatic encephalopathy (HE). The clinical manifestations of HE range from the mildly altered level of sensorium to severely altered consciousness levels, difficulty in judgment, the day-night reversal of sleep, flapping tremor of hands, and irrelevant talking or speech. Patients with hyponatremia are at a higher risk of developing HE and electroencephalographic abnormalities. The severity of hyponatremia is directly related to the deterioration in terms of grades of HE. Our study sought to determine the frequency of hyponatremia in cirrhotic patients and its correlation with the frequency and severity of HE.

Methodology

This study was carried out at the inpatient department of medicine in a tertiary care hospital in Pakistan. A total of 260 patients of both genders with hepatic cirrhosis were approached. After taking relevant history and physical examination, the venous blood sample of each patient was drawn and sent to the institutional laboratory for estimation of serum electrolytes, liver function tests (LFTs), renal parameters (RPMs), prothrombin time (PT), activated partial thromboplastin time (aPTT), and international normalized ratio (INR). We classified the HE according to the West Haven classification system. Mild to moderate encephalopathy was classified under grades I-II, while severe encephalopathy was classified under grades III-IV. We documented the severity of liver disease according to the Child-Pugh score criteria. All data were analyzed by using SPSS Statistics version 25.0 (IBM, Armonk, NY). We reported the data as means along with the standard error.

Results

Overall, the serum sodium levels of the subjects ranged from 115 to 142 meq/L with a mean of 129.11 ±6.53 meq/L. In patients with hyponatremia, it ranged from 115 to 127 meq/L (mean 121.41 ±5.17 meq/L). Hyponatremia was present in 96 (36.9%) patients. Among these, 51 (53.12%) were male and 45 (46.8%) were female; 24 (9.2%) patients had mild hyponatremia, 56 (21.5%) had moderate, and 16 (6.2%) had severe hyponatremia. HE was present in 176 (67.7%) patients. HE grade I was present in 54 (20.8%), grade II in 62 (23.8%), grade III in 32 (12.3%), and grade IV in 28 (10.8%) patients. In 96 patients with hyponatremia, 84 were found to have HE (p-value: <0.001).

Conclusion

Based on our findings, cirrhotic patients with chronic hepatitis infections have a variable presence of low sodium levels. Sodium levels of <130 meq/L were associated with higher morbidity and mortality rate. Moreover, patients with lower levels of sodium had higher grades of HE.

## Introduction

Hepatic cirrhosis is one of the leading causes of morbidity and mortality worldwide [1]. It is a very common medical problem in underdeveloped countries, resulting in a heavy burden on the healthcare system and health professionals. Patients with cirrhosis frequently develop complications like ascites, variceal bleeding, and hepatic encephalopathy (HE) [2]. The annual rate of growth of HE in cirrhotic patients is about 8% [3]. Various underlying pathologies like constipation, esophageal variceal bleed, and infections like spontaneous bacterial peritonitis can precipitate HE [3].

The clinical manifestations of HE range from the mildly altered level of sensorium to severely altered consciousness levels, difficulty in judgment, the day-night reversal of sleep, flapping tremor of hands, and irrelevant talking or speech [4]. Similarly, the patients with liver cirrhosis experience disturbance in the regulation of body fluid homeostasis. The kidneys start to retain the water excessively, which results in a significant derangement of sodium levels in the serum [5]. It is evident that the chances of developing HE and electroencephalographic abnormalities are high in the presence of hyponatremia.

The underlying mechanism that precipitates hepatic encephalopathy as a result of hyponatremia is not fully understood. It has been postulated that the formation of an osmotic gradient between extracellular fluid compartments leads to the swelling of astrocytes [6]. Hyponatremia is an indicator of the worsening of the disease and increases the risk of HE by about eightfold [7]. The severity of hyponatremia is directly related to the deterioration in terms of the grades of HE [8].

Recent studies have concluded that hyponatremia is a key prognostic factor in patients with chronic liver disease. Moreover, the patients with hyponatremia have poor survival rates as compared to those without hyponatremia [5,9]. Our study sought to determine the frequency of hyponatremia in cirrhotic patients and its correlation with the frequency and severity of HE. We believe that the findings of our study will aid in developing new strategies or modification of existing guidelines regarding the management of cirrhotic patients with hyponatremia and HE.

## Materials and methods

Study setting

This study was carried out at the inpatient department of medicine in a tertiary care hospital in Multan, Pakistan.

Subjects, sample size, and sampling technique

A total of 260 patients of both genders with hepatic cirrhosis were approached. Simple random sampling was performed.

Study design

The research approach employed a cross-sectional study design to correlate hyponatremia with HE and the severity of the liver disease.

Inclusion and exclusion criteria

Patients with diagnosed hepatic cirrhosis of any etiology who were aged between 20-80 years were included in this study. Patients under treatment for hepatocellular carcinoma, ascites, and hyponatremia were excluded. Patients with underlying renal pathology, those on dialysis, and patients on diuretic therapy were also excluded.

Data collection procedure

After obtaining approval from the ethical committee of the hospital, the study was carried out in the inpatient department of internal medicine. All the patients were informed about the nature of the study and their verbal consent was obtained. After taking relevant history and physical examination, the venous blood sample of each patient was drawn and sent to the institutional laboratory for estimation of serum electrolytes, liver function tests (LFTs), renal parameters (RPMs), prothrombin time (PT), activated partial thromboplastin time (aPTT), and international normalized ratio (INR). We classified the HE according to the West Haven classification system. Mild to moderate encephalopathy was classified under grades I-II, while severe encephalopathy was classified under grades III-IV. We documented the severity of liver disease according to the Child-Pugh score criteria. The patients were classified into different groups based on the serum sodium concentration as follows: level of <130 meql/l (significant/severe hyponatremia), between 131 and 135 meq/l (mild hyponatremia), and level of >135 meq/l (normal). A specialized proforma was designed to collect all the study information.

Data analysis

All data were analyzed using SPSS Statistics version 25.0 (IBM, Armonk, NY). We reported the data as means along with standard error. Frequencies and percentages were calculated for gender, hyponatremia, and the presence of HE. Chi-square test and Spearman's rank test were employed to correlate hyponatremia with HE and its severity. A p-value of <0.05 was considered statistically significant. Conclusions were drawn accordingly.

## Results

Among the patients, there were 185 (75%) males and 75 (25%) females; the mean age of the patients was 53.7 ±11.3 years (range: 35-80 years). Overall, serum sodium levels among the subjects ranged from 115 to 142 meq/L, with a mean of 129.11 ±6.53 meq/L. In patients with hyponatremia, it ranged from 115 to 127 meq/L (mean: 121.41 ±5.17 meq/L).

Hyponatremia was present in 96 (36.9%) patients. Among these, 51 (53.12%) were male and 45 (46.8%) were female; 24 (9.2%) patients had mild hyponatremia, 56 (21.5%) had moderate, and 16 (6.2%) had severe hyponatremia.

HE was present in 176 (67.7%) patients. HE grade I was present in 54 (20.8%), grade II in 62 (23.8%), grade III in 32 (12.3%), and grade IV in 28 (10.8%) patients. In 96 patients with hyponatremia, 84 were found to have HE (p-value: <0.001). The association of hyponatremia with HE is shown in Table 1.

**Table 1:** Hyponatremia and its correlation with hepatic encephalopathy (n=260)

Hyponatremia	Hepatic encephalopathy		Total	Significance
	Yes	No		
Yes	84	12	96	P-value: <0.001
No	92	72	164	
Total	176	84	260	

Table 2 shows the correlation of the severity of hyponatremia with grades of HE (p-value: <0.001).

**Table 2:** Correlation of severity of hyponatremia with grades of hepatic encephalopathy (n=260)

Severity of hyponatremia	Grades of hepatic encephalopathy					Total	Significance
	I	II	III	IV	None		
Mild	8	6	4	2	4	24	P-value: <0.001
Moderate	22	18	6	2	8	56	
Severe	2	2	6	6	0	16	
None	22	36	16	18	72	164	
Total	54	62	32	28	84	260	

## Discussion

Chronic infection with hepatitis B and C can be asymptomatic or can be associated with inflammation of the liver, leading to cirrhosis [10]. Every year, more than one million patients with chronic viral hepatitis infection die because of complications such as cirrhosis and hepatic carcinoma [11]. Hyponatremia is a well-known complication of liver failure. It occurs due to fibrosis and leads to increased cause-specific mortality and a significantly high number of hospital admissions.

In our study, hyponatremia was present in 96 (37%) patients; serum sodium levels among the patients ranged from 115 to 127 meq/L, with a mean of 121.41 ±5.17 meq/L. Our results are consistent with those of previous studies. In a study by Khalil et al., it was shown that the prevalence of hyponatremia (serum sodium level of <130 meq/L) was 45.5% among the cohort, with a mean of 123.26 ±5.57 meq/L [12]. In another study, the prevalence of hyponatremia was found to be 30% [13]. A study done by Jenq et al. revealed that cirrhotic patients with hyponatremia had a higher in-hospital mortality rate [14].

In our study, HE was present in 176 (67.7%) patients; and 82 out of 96 (87.5%) patients with hyponatremia had HE (p-value: <0.001). In a study conducted by Udagani et al., it was revealed that cirrhotic patients with hyponatremia had a greater risk of developing neurological disorders as compared to those who had normal sodium levels [15]. Similarly, in the above-mentioned study, the risk of developing HE was found to be 2.8 fold greater in patients with low sodium levels [12]. Hence, the evidence clearly indicates that hyponatremia may affect brain function and predispose patients to HE.

Our results showed that the correlation of severity of hyponatremia with grades of HE was statistically significant (p-value: <0.001). Our findings are in line with those of previous studies. In a study by Angeli et al., it was reported that 38% of the severe hyponatremic patients had HE as compared to 24% of patients with mild hyponatremia [8].

The risk of developing ascites, variceal bleeding, HE, and other cirrhosis-related complications is directly proportional to the degree/severity of hyponatremia. Various studies have shown that severe hyponatremia is associated with increased severity of HE [9,16,17]. The association between HE and hyponatremia may be explained based on the higher severity of liver disease among patients with sodium levels of <130 meq/L and the hypothesis that there might be a pathophysiological link between these two events. In a study by Cordoba et al., it was concluded that hyponatremia causes mild cerebral edema, which results in increased osmotic pressure on astrocytes. Eventually, it leads to many neurological dysfunctions [18].

Our study has some limitations. Firstly, the result might be biased due to the open-label design of the study. Secondly, the lab values for sodium can be affected by human or machine errors. Thirdly, only clinicians assessed the patients for clinical findings. Finally, the study consisted of a relatively small population. Further longitudinal studies with larger populations are needed to more comprehensively analyze and identify the correlation of hyponatremia with the severity of liver disease and HE.

## Conclusions

Our study concluded that cirrhotic patients with chronic hepatitis infections have a variable presence of low sodium levels. Patients with lower levels of sodium had higher grades/severity of HE. Cirrhotic patients with hyponatremia should be managed very carefully as it can lead to various neurological complications.

## References

[1] Scaglione S, Kliethermes S, Cao G, Shoham D, Durazo R, Luke A, Volk ML (2015). The epidemiology of cirrhosis in the United States: a population-based study. J Clin Gastroenterol.

[2] Memon AR, Shafique K, Memon A, Draz AU, Rauf MU, Afsar S (2012). Hepatitis B and C prevalence among the high risk groups of Pakistani population. A cross sectional study. Arch Public Health.

[3] Mumtaz K, Ahmed US, Abid S, Baig N, Hamid S, Jafri W (2010). Precipitating factors and the outcome of hepatic encephalopathy in liver cirrhosis. J Coll Physicians Surg Pak.

[4] Liu A, Perumpail RB, Kumari R, Younossi ZM, Wong RJ, Ahmed A (2015). Advances in cirrhosis: optimizing the management of hepatic encephalopathy. World J Hepatol.

[5] Shaikh S, Mal G, Khalid S, Baloch GH, Akbar Y (2010). Frequency of hyponatraemia and its influence on liver cirrhosis-related complications. J Pak Med Assoc.

[6] Tivers MS, Handel I, Gow AG, Lipscomb VJ, Jalan R, Mellanby RJ (2014). Hyperammonemia and systemic inflammatory response syndrome predicts presence of hepatic encephalopathy in dogs with congenital portosystemic shunts. PloS One.

[7] Yun BC, Kim WR (2009). Hyponatremia in hepatic encephalopathy: an accomplice or innocent bystander?. Am J Gastroenterol.

[8] Angeli P, Wong F, Watson H, Ginès P; CAPPS Investigators (2006). Hyponatremia in cirrhosis: results of a patient population survey. Hepatology.

[9] Cárdenas A, Ginès P (2008). Predicting mortality in cirrhosis--serum sodium helps. N Engl J Med.

[10] Shakeel HA, Maqsood H, Ali B, Khan AR (2018). Association of chronic viral hepatitis with ABO blood groups and rhesus (Rh) factor. Int J Res Med Sci.

[11] Khan D, Shakeel HA, Maqsood H, Aziz HMA, Qazi MJ, Shah SAY (2018). Immunization status of students of Nishtar Medical University against hepatitis B. Int J Res Med Sci.

[12] Khalil OA, Abdel-Aziz A, El-okely AM, Mikheil NG, Al-Nahal S (2013). Prevalence of hyponatremia and its association with development and severity of complications in cirrhotic patients. Zagazig Univ Med J.

[13] Borroni G, Maggi A, Sangiovanni A, Cazzaniga M, Salerno F (2000). Clinical relevance of hyponatraemia for the hospital outcome of cirrhotic patients. Dig Liver Dis.

[14] Jenq CC, Tsai MH, Tian YC (2010). Serum sodium predicts prognosis in critically ill cirrhotic patients. J Clin Gastroenterol.

[15] Udagani P, Vibha C, Vishwanath HL (2013). The association between hyponatremia and severity of complications in liver cirrhosis. Int J Curr Res.

[16] Barakat AA, Metwaly AA, Nasr FM, El-Ghannam M, El-Talkawy MD, Taleb HA (2015). Impact of hyponatremia on frequency of complications in patients with decompensated liver cirrhosis. Electron Physician.

[17] Guevara M, Baccaro ME, Ríos J (2010). Risk factors for hepatic encephalopathy in patients with cirrhosis and refractory ascites: relevance of serum sodium concentration. Liver Int.

[18] Cordoba J (2014). Hepatic encephalopathy: from the pathogenesis to the new treatments. ISRN Hepatol.

